# TRACKING DOWN ALPHA-PARTICLES: THE DESIGN, CHARACTERISATION AND TESTING OF A SHALLOW-ANGLED ALPHA-PARTICLE IRRADIATOR

**DOI:** 10.1093/rpd/ncy300

**Published:** 2019-02-06

**Authors:** James M Thompson, Amy Elliott, Sofia D’Abrantes, Gabriel O Sawakuchi, Mark A Hill

**Affiliations:** 1 CRUK/MRC Oxford Institute for Radiation Oncology, University of Oxford, Gray Laboratories, ORCRB Roosevelt Drive, Oxford OX3 7DQ, UK; 2 Department of Radiation Physics, The University of Texas MD Anderson Cancer Center, 1400 Pressler St., Unit 1420, Houston, TX 77030, USA

## Abstract

Human exposure to *α*-particles from radon and other radionuclides is associated with carcinogenesis, but if well controlled and targeted to cancer cells, *α*-particles may be used in radiotherapy. Thus, it is important to understand the biological effects of *α*-particles to predict cancer risk and optimise radiotherapy. To enable studies of *α*-particles in cells, we developed and characterised an *α*-particle automated irradiation rig that allows exposures at a shallow angle (70° to the normal) of cell monolayers in a 30 mm diameter dish to complement standard perpendicular irradiations. The measured incident energy of the *α*-particles was 3.3 ± 0.5 MeV (LET in water = 120 keV μm^−1^), with a maximum incident dose rate of 1.28 ± 0.02 Gy min^−1^, which for a 5 μm cell monolayer corresponds to a mean dose rate of 1.57 ± 0.02 Gy min^−1^ and a mean LET in water of 154 keV μm^−1^. The feasibility of resolving radiation-induced DNA double-strand breaks (DSB) foci along the track of *α*-particles was demonstrated using immunofluorescent labelling with γH2AX and 53BP1 in normal MRC-5 human lung cells.

## INTRODUCTION

Alpha-particles from radon and its progeny contribute approximately to 50% of the annual effective dose to the UK population, but the concentration of radon can vary by many orders of magnitude depending on location. Additionally, there is increasing interest in the clinical use of *α*-particle radionuclides to treat a variety of cancers, including radionuclides conjugated with monoclonal antibodies developed to directly target tumour cells^(1)^. Thus, it is important to understand the mechanism of *α*-particles interaction with biological systems to accurately predict cancer risk and optimise radiotherapy.

These *α*-particles have high ionisation densities and the energy transferred per unit distance [or linear energy transfer (LET)] is high compared to low-LET radiation such as X-rays and *γ*-rays. *α*-particles are typically emitted with energies ranging from 5 to 8 MeV, corresponding to ranges in tissue from 37–77 μm. As an *α*-particle slows down, its LET increases from ~70 to 90 keV μm^−1^ at the start of the track to a peak of ~237 keV μm^−1^ towards the end of the track before falling again at the very end of its range. In comparison to low-LET radiation, *α*-particles have a higher relative biological effectiveness (RBE) at inducing a range of biological end-points^(2)^, including cell inactivation^(3–5)^, mutation induction^(6, 7)^ and transformation^(8)^. The RBE increases with increasing LET up to a peak around 100 keV μm^−1^ and then decreases at higher LET values^(3, 5)^. For example, for 3.2 MeV *α*-particles incident on V79-4 cells (average LET of 131 keV μm^−1^ across the cell) a maximum low-dose RBE (RBE_M_) of 10.2 ± 0.2 was reported^(5)^. The high RBE of *α*-particles is due to its densely ionising track structure^(9, 10)^, which induces clustered DNA damage (two or more lesions within one or two helical turns of DNA). This includes DNA double-strand breaks (DSB) and complex DSB (consisting of simple DSB with additional strand breaks and/or base damage within the cluster). Monte Carlo modelling shows that the yield of DSB which are complex is ~90%, for *α*-particles, compared to ~30–50% for low-LET radiation (e.g. X-rays and *γ*-rays)^(11, 12)^. These complex DSB result in decreased DSB repair rate and increased residual DSB yield^(13)^.

In addition to the high efficiency at inducing complex DSB, *α*-particles also produce spatially and temporally correlated DSB along the narrow track of the particle (maximum range of δ-electrons typically <0.1 μm, with ~90% of energy deposition within 10 nm)^(14)^. This occurs in individual chromosomes within the nucleus (e.g. in DNA around nucleosomes and chromatin fibre/loops) and between separate chromosomes occupying adjacent territories^(15, 16)^. The close proximity of these breaks increases the probability of illegitimate re-joining producing chromosomal rearrangements. As a result, the passage of a single *α*-particle is efficient at producing complex chromosome aberrations (requiring three or more breaks in two or more chromosomes), in contrast to mainly simple aberrations (maximum of two breaks in two chromosomes) observed for low doses of low-LET X-rays^(16, 17)^.

Biological effects of *α*-particles have been studied with conventional irradiation of a cell monolayer with a perpendicular beam (relative to the dish) of *α*-particles^(5, 18, 19)^. However, it is difficult to resolve surrogates for DNA lesions, such as foci, because of the diffraction-limited resolution of conventional microscopes—where spatial resolution is even poorer in the z-axis. This low-resolution in the *z*-axis limits the study of DNA repair kinetics. To allow using the higher spatial resolution in the focal plane (*x* – *y*) of conventional microscopes, we modified the existing Oxford *α*-particle irradiator to enable shallow angle irradiations of cell monolayers. We further validated our setup with initial immunofluorescence studies demonstrating the improved spatial resolution of foci along *α*-particle tracks.

## METHODS

### Shallow-angled *α*-particle irradiator rig

**Figure 1.**
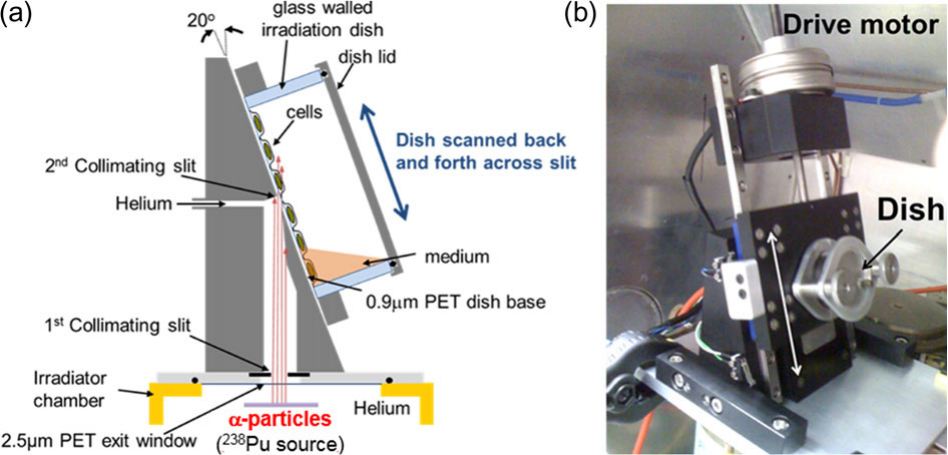
Shallow-angled *α*-particle rig positioned above the exit window of the Oxford *α*-particle irradiator: (**a**) schematic (not to scale) and (**b**) photograph with double-headed arrow showing direction of motion.

The shallow-angled irradiation rig enables irradiation of cell monolayers at a 70° angle to the normal by scanning custom made irradiation dishes across a collimated *α*-particle beam (~4 mm × 30 mm) at the required angle. These glass-walled irradiation dishes (30 mm internal diameter) incorporate a 0.9 μm PET (polyethylene terephthalate; DuPont Teijin films, Dumfries, UK) base to minimise energy loss of traversing *α*-particles. The design of the rig is illustrated in Figure 1 and was built to attach to the top of the existing Oxford *α*-particle irradiator^(18)^, with the source raised so that the emitted *α*-particles traverse 10 mm in helium prior to exiting the 2.5 μm PET window and a subsequent 54 mm in helium to the 0.9 μm PET base of the irradiation dish at the centre of the 4 mm wide final (second) collimating slit (i.e. a source to cell distance of 64 mm). The plane of this slit is at a 20° angle to the normal of the 1 GBq ^238^Pu source and parallel to the dish base. Both the original irradiator chamber and the inside of the angled-irradiator rig were continuously flushed with helium at atmospheric pressure. The irradiation dish is scanned across the angled slit using a stepper motor, with the limit switches used to define the range of motion. An in-house built controller was used to set the total number of traversals, with each traversal (from one limit switch to the other) taking 5.6 s. An O-ring seal ensures that the helium environment is maintained under the dish as it is scanned, along with a PTFE gasket between the moving plate holding the dish and the static plate holding the slit. The response following shallow-angle exposures was compared to the response of cells irradiated perpendicular to the base with 3.26 MeV (LET in water of 121 keV μm^−1^) *α*-particle using the standard Oxford irradiator^(5, 18)^.

### Fluence, energy and dose measurements

The total fluence of *α*-particles and therefore the dose delivered to the dish was ultimately determined by the number of traversals of the dish across the slit. However, the fluence per traversal could also be varied by selecting the aperture directly above the ^238^Pu source (25, 4.5. 1.4 or 0.5 mm diameter; the 25 mm aperture is larger than the source with a diameter of ~20 mm) and the width of first collimating slit (currently either 1 mm or 7.5 mm wide; 30 mm long).

The fluence of *α*-particles across the scanned dish was determined using 25 mm diameter fluorescence nuclear track detector (FNTD) discs (Landauer Inc., Stillwater, OK, USA)^(20)^ placed directly on the PET base at the centre of the irradiation dish. The FNTDs were subsequently exposed by traversing the dish over the slit five times for the 7.5 mm wide first collimating slit and 50 times for the 1 mm wide first collimating slit. Following irradiation, the resulting tracks were imaged with a Zeiss LSM 710 confocal microscope using a 63×/1.4 oil objective and a 5 mW HeNe laser (excitation 633 nm, collection 634 nm–755 nm). The 1024 × 1024 pixel (135 × 135 μm^2^) images were averaged over eight collections per slice with a dwell time of 3.15 μs pixel^−1^ per collection.

Energy measurements were performed using an A300-17AM Passivated Implanted Planar Silicon (PIPS) surface barrier detector (Canberra Industries Inc., Meriden, CT, USA) coupled to an alpha spectrometer comprised of a Model *2003BT* charge sensitive FET input pre-amplifier and a DSA-1000 multichannel analyser (Canberra Industries Inc., Meriden, CT, USA). The detector and spectrometer were calibrated using a three peak (^239^Pu, ^241^Am and ^244^Cm) calibration source (Isotrak QCRB2508, AEA technology QSA, Didcot, UK) in a vacuum chamber. The three dominate peaks produced with the detector at the same angle as the dish correspond to *α*-particle energies of 5.156 MeV, 5.486 MeV and 5.805 MeV with respective full width half maximum (FWHM) values of 18 keV, 14 keV and 13 keV. Measurements were performed with the surface barrier detector directly behind the PET base of a sample irradiation dish, flushed with helium.

As the *α*-particles traverse the cell and lose energy, there is a corresponding increase in LET. Therefore, the mean dose to the cell is different to the incident dose to the cell at the PET-cell interface and is dependent on distribution of cell thicknesses. The average dose to the cell monolayer at a given depth for *α*-particles incident on the cell at an angle θ to the normal of the dish base can be calculated using:

(1) D=ΦL/(ρcosθ)

where *ρ* is the density, Φ is the particle fluence and *L* is the LET in water [this corresponds to *D* (Gy) = 0.16 × Φ (particles μm^−2^) × *L* (keV μm^−1^)/cos θ, assuming a cell density of 1 g cm^−3^]. For a given incident energy, *E*_*i*_, the variation in energy and LET along the remaining path of the *α*-particle as it slows down can be determined using SRIM stopping power data for helium ions in water^(21, 22)^. Equation (1) can then be used to calculate the relative variation in dose per incident particle of energy *E_i_* as a function depth in a cell monolayer of thickness, *t*. An estimate of the average dose to the cell monolayer was calculated from the measured total fluence, Φ, and numerically integrated across the measured incident energy spectrum, for a 5 μm cell monolayer thickness^(5)^.

### Cell culture

MRC-5 human foetal lung fibroblast cells were cultured in minimum essential medium (MEM) supplemented with 10% FBS (foetal bovine serum), 1% NEAA (non-essential amino acids), 100 units ml^−1^ penicillin (Sigma-Aldrich), 100 μg ml^−1^ streptomycin (Sigma-Aldrich) and 2 mM L-Glutamine (Sigma-Aldrich). Cells were incubated at 37°C with 5% CO_2_ humidified air. Approximately 1 day prior to irradiation, 3 × 10^5^ cells in 2 ml were plated in irradiation dishes to produce an attached cell monolayer on the PET dish base.

### Immunofluroescent labelling of DSB

Following irradiations, cells were incubated for 30 min then washed with phosphate-buffered saline (PBS) (Thermo Fisher Scientific), fixed in 1 ml 4% paraformaldehyde solution (Sigma-Aldrich) for 30 min at 4°C, then washed three times in PBS prior to storing at 4°C. Each dish was subsequently treated with 1 mL permeabilising buffer (0.25% Triton X-100 (Sigma-Aldrich) in PBS) for 5 min at room temperature, washed three times with PBS prior to incubating in 1 mL blocking buffer (1% BSA, bovine serum albumin (Sigma-Aldrich), in PBS) for 15 min at room temperature. The cells were then incubated in the primary antibody solution for 45 min at room temperature. The primary antibody solution consisted of 1 μg mouse anti-γH2AX antibody (Merck Millipore) and 1 μg rabbit anti-53BP1 (Bethyl Laboratories) made up to 500 μl in blocking buffer. Samples were then washed three times with PBS, before incubating in secondary antibody solution for 30 min in the dark at room temperature. The secondary antibody solution consisted of 1 μg AlexaFluor 488 donkey anti-mouse (Thermo Fisher Scientific) and 1 μg AlexaFluor 633 anti-rabbit (Thermo Fisher Scientific) made up to 500 μL in blocking buffer. Samples were finally washed three times with PBS in the dark prior to adding Vectashield Mounting Medium containing DAPI (Vector Laboratories Ltd.) and covering with a 22 mm diameter glass coverslip. The samples were stored in the dark at 4°C. Imaging was performed using a Zeiss LSM 800 confocal scanner with a 63x oil objective.

## RESULTS

### Fluence, energy and dose measurements

**Figure 2.**
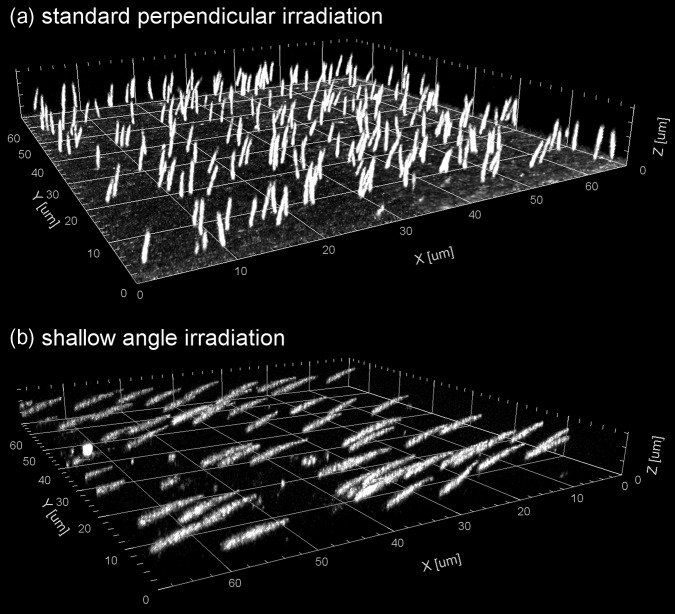
3D image of *α*-particle tracks measured using FNTD: (**a**) for standard perpendicular irradiation with a corresponding dose of 1 Gy; (**b**) for angled irradiation after 50 traversals of the slit (using a 1 mm wide first collimating slit).

**Figure 3.**
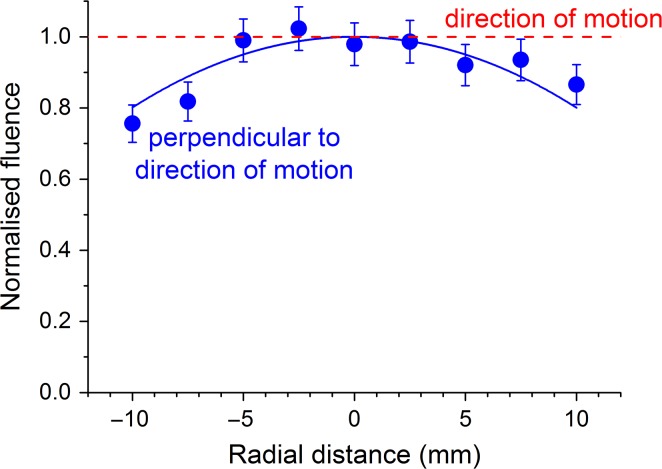
Variation in *α*-particle fluence across the sample dish perpendicular to the direction of motion measured using FNTD. (fitted solid line: Φ(r) = 1 – 0.00199 *r*^2^). As a result of the dish travelling at a constant speed across the slit the fluence will be constant in the direction of motion (dashed line).

FNTDs were used to image individual *α*-particle tracks for a standard perpendicular irradiation and shallow-angled irradiations (Figure 2). For the shallow-angled irradiations, the measured *α*-particle fluence per traversal at the centre of the dish was 0.31 ± 0.02 × 10^−3^ μm^−2^ and to 2.1 ± 0.2 × 10^−3^ μm^−2^ for the 1.0 mm and 7.5 mm wide first collimator, respectively. The variation in *α*-particle fluence across the sample dish perpendicular to the direction of motion for shallow-angled irradiation after 50 traversals using a 1 mm wide first collimating slit is shown in Figure 3, with the fitted normalised distribution given by the equation *f*(*x*) = 1 – 0.00199 *x*^2^. However, the fluence is constant in the direction of motion. In order to calculate the average fluence over the circular dish base (of radius, *r* = 15 mm). the distribution across the length of the static slit, *f*(*x*), was integrated across the dish using the equation:

(2) $$\int_{-r}^{r}\frac{2f\left(x\right)\sqrt{r^{2}-x^{2}}dx}{\pi r^{2}}$$

Therefore, the average fluence corresponds to 0.89 times the fluence at the centre of the dish.

**Figure 4.**
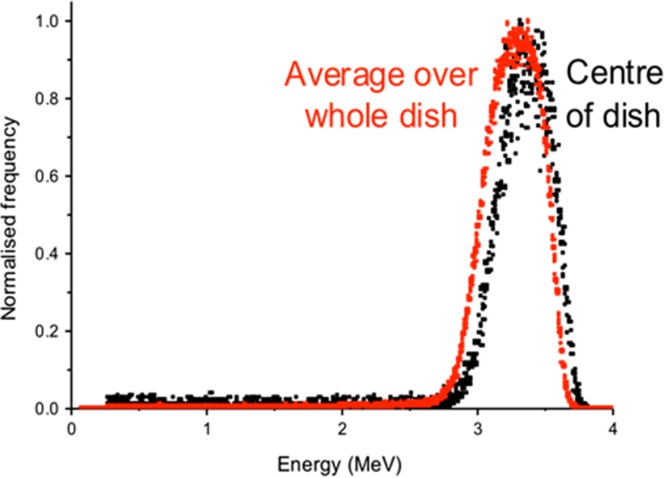
Measured energy spectra of the *α*-particles incident on the cells (after passing through the PET base of the sample dish).

The measured energy spectra of the *α*-particles incident on the cell monolayer (after passing through the PET base of the irradiation dish) at the centre of the dish had a peak energy of 3.4 MeV and a FWHM of 0.5 MeV (Figure 4). There is a slight reduction in energy when averaged over the majority of dish (represented by the 300 mm^2^ active area of the surface barrier detector) with peak energy of 3.3 MeV (FWHM = 0.5 MeV) (Figure 4).

The incident dose rate at the centre of the dish for a 1 and 7.5 mm wide first collimator was 0.19 Gy min^−1^ and 1.28 Gy min^−1^, respectively (Table 1). The corresponding mean dose rates were 0.23 Gy min^−1^ and 1.57 Gy min^−1^ for a 5 μm thick cell monolayer, respectively. A more accurate assessment of the dose rates across the dish can be made by including the variation in angle of incident, range straggling and the variation in cell geometry.

**Table 1.** Summary of -particle energy, fluence per traversal, incident dose rate and mean dose rate to a 5 μm cell monolayer (5.6 s per traversal) obtained using the 1 mm wide first collimator.

	Centre of dish
Peak energy	3.4 ± 0.5 MeV
Fluence per traversal	0.31 ± 0.03 × 10^−3^ μm^−2^
Incident surface dose rate	0.19 ± 0.02 Gy min^−1^
Incident LET	123 keV μm^−1^
Mean dose rate (5 μm thick)	0.23 ± 0.02 Gy min^−1^
Mean LET	152 keV μm^−1^

### Visualisation of *α*-particle tracks

**Figure 5.**
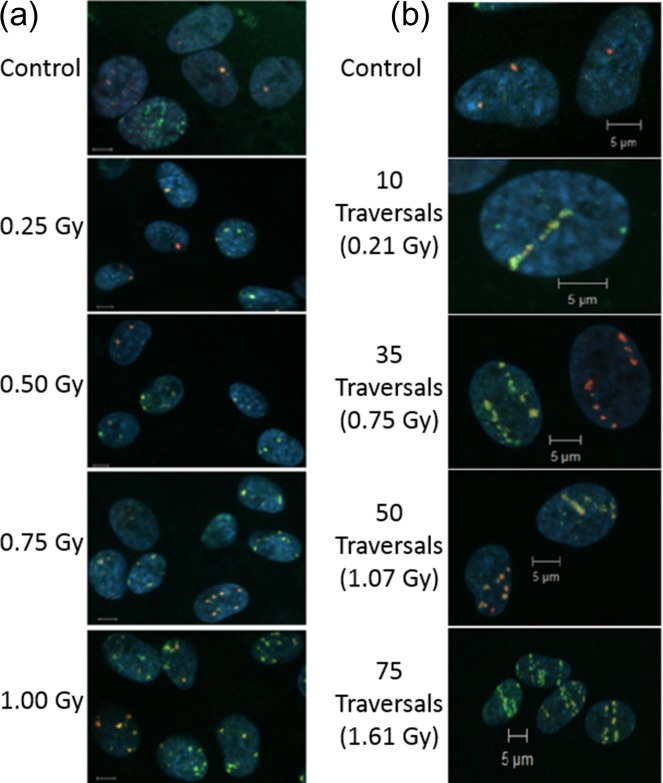
Induction of γH2AX (green) and 53BP1 foci (red) in the nucleus (blue) of MRC-5 cells induced following (**a**) perpendicular irradiation and (**b**) shallow-angled irradiation with *α*-particles (cell nuclei are labelled in blue using DAPI staining). Scale bar, 5 μm.

We exposed MRC-5 cells to both standard perpendicular or shallow-angled irradiations *α*-particles to validate that γH2AX and 53BP1 foci can be observed. As expected, the number of foci increases with increasing fluence. While with the perpendicular irradiations, it is difficult to resolve individual foci along the track, the shallow-angled irradiations clearly show multiple foci along the path of the *α*-particles traversing the nucleus (Figure 5). A number of the resulting foci-tracks do not appear to traverse the whole nucleus, however due to the angle of incidence of the particle, these typically represent tracks either entering the nucleus from below or exiting from the top.

## CONCLUSIONS

To complement standard perpendicular *α*-particle irradiations, a new automated irradiation rig has been developed to expose mammalian cells in a 30 mm diameter dish to *α*-particles at shallow-angles (70° to the normal). The measured incident energy of the *α*-particles was 3.3 ± 0.5 MeV (LET = 120 keV μm^−1^) at the centre of the dish for a 1 mm wide first collimator. This corresponded to an incident dose rate of 0.19 ± 0.02 Gy min^−1^ and a mean dose rate of 0.23 ± 0.02 Gy min^−1^ for 5 μm thick cell monolayer, with a mean LET of 154 keV μm^−1^. The mean dose rate could be increased to 1.57 Gy min^−1^ if a 7 mm first collimator slit is used. Lower dose rates are achievable by reducing the size of the aperture in front of the ^238^Pu source. The immunofluorescence studies performed clearly demonstrate the ability of these shallow-angled irradiations to resolve sites of damage along the track of the *α*-particle and therefore facilitating DNA repair studies. In addition to investigating the induction and repair of DSB, these techniques can also be used to study the kinetics of recruitment and loss of DNA repair proteins in wild type and repair deficient cells. The use of live cell imaging of fluorescently tagged proteins can also be used to help shed light on the spatial dynamics of these breaks post exposure. Due to the high ionisation density along the path of the *α*-particle, it is still likely that not all DSB are resolved. Therefore, it would be interesting to use a super-resolution microscopy to explore the structure of the foci. In addition to DNA repair studies, the ability to irradiate a 30 mm diameter dish also enables the effect of cell geometry (with respect to the track) to be explored by comparing differences in biological response to shallow-angled *α*-particle exposure to and an identical dose delivered perpendicular to the cell monolayer.

## FUNDING

The authors gratefully acknowledge funding from Medical Research Council Strategic Partnership (MC_PC_12004) for the CRUK/MRC Institute for Radiation Oncology.
